# Internal Characterization-Based Prognostics for Micro-Direct-Methanol Fuel Cells under Dynamic Operating Conditions

**DOI:** 10.3390/s22114217

**Published:** 2022-06-01

**Authors:** Dacheng Zhang, Xinru Li, Wei Wang, Zhengang Zhao

**Affiliations:** 1 Faculty of Information Engineering and Automation, Kunming University of Science and Technology, Kunming 650500, China; dacheng.zhang@kust.edu.cn (D.Z.); lixinru@stu.kust.edu.cn (X.L.); 2 Yunnan Key Laboratory of Green Energy, Electric Power Measurement Digitalization, Control and Protection, Kunming 650500, China; 3 Department of Mechanical Engineering, City University of Hong Kong, Hong Kong 999077, China; wwang326@cityu.edu.hk

**Keywords:** micro-direct-methanol fuel cell, internal characterization, prognostics, operating conditions, remaining useful life

## Abstract

Micro-direct-methanol fuel cells (μDMFCs) use micro-electro mechanical system (MEMS) technology, which offers high energy density, portable use, quick replenishment, and free fuel reforming and purification. However, the μDMFC is limited by a short effective service life due to the membrane electrode’s deterioration in electrochemical reactions. This paper presents a health status assessment and remaining useful life (RUL) prediction approach for μDMFC under dynamic operating conditions. Rather than making external observations, an internal characterization is used to describe the degradation indicator and to overcome intrusive influences in operation. Then, a Markov-process-based usage behavior prediction mechanism is proposed to account for the randomness of real-world operation. The experimental results show that the proposed degradation indicator alleviates the reduction in μDMFC output power degradation behavior caused by the user loading profile. Compared with the predictions of RUL using traditional external observation, the proposed approach achieved superior prognostic performance in both accuracy and precision.

## 1. Introduction

Due to the increased popularity and extended applications of portable electronic products, such as notebook computers and cell phones, power supply systems demand higher performance requirements in usage scenarios. Traditional power supplies have gradually failed to meet technological development needs. Micro-electro mechanical systems (MEMSs) have significant advantages in size, mass, energy density, and cost, etc., meaning that they can solve the energy supply problem that currently limits the development of micro-electronic products [1]. As a new type of micro-energy with broad application prospects, micro-direct-methanol fuel cells (μDMFCs) have significant advantages, such as the abundance and low price of methanol, easy storage and portability, safety, simple system structure, and their absence of fuel reforming or purification requirements. They are suitable for portable electronic products and micro-weapon systems, and are a hot topic in the field of micro-fuel-cells [2,3].

μDMFC belongs to the proton exchange membrane fuel cell (PEMFC), which features a complex multi-physics and multi-scale system [4]. The development of DMFC is due to the emphasis on improving the materials used and modifying its structure to increase efficiency [5,6,7]. However, the high maintenance cost and limited service life also limit its commercial viability [8]. The prognostics and health management (PHM) approach dynamically manages a system’s life duration [9,10,11], enabling reliability evaluations to made in its current condition, predicting failures and mitigating the risk of malfunctioning. Prognostics results can inform decisions on maintenance scheduling and control the strategies that minimize maintenance time, extend service life, improve durability, and avoid catastrophic failures of the μDMFC [12].

Research on μDMFC prognostics is scarce, and few analytical models have been used to describe its degradation mechanism [13,14]. Due to the complexity of the system, data-driven methods have attracted increasing amounts of attention. He et al. [15] used a back propagation neural network (BPNN) and an adapted neural fuzzy to estimate the fuel cell performance in both stationary and nonstationary conditions. Meraghni et al. [16] developed a data-driven digital twin (DT) prognostics approach to predict the RUL of the PEMFC. Despite the multiple advantages, such as physical modeling being unnecessary, low computation cost, and easy implementation, data-driven methods lack accuracy when describing degradation mechanisms. However, the modeling of the μDMFC degradation mechanism is of great importance for capturing the three critical aging phenomena during their operations: ohmic loss, activation loss, and mass transfer loss. Fang et al. [17] evaluated the effect of various operating parameters on the behavior of the DMFC stack using a systemic model. In the work of Cheng et al. [18], the activation loss under different loads was accurately obtained by solving the implicit Butler Volmer equation. Ismail et al. [19] presented a 2D multiphase non-isothermal mass transfer model for a single-cell DMFC, which laid the foundation for DMFC RUL prediction. Zhou et al. [20] adopted a new approach based on a multi-physical aging model to predict the voltage outputs of a fuel cell. Those studies delivered promising results even when the available training data are limited. They laid the foundation for studying degradation parameters, supporting μDMFC degradation mechanism modeling and RUL prediction.

However, the models have been only verified under a constant current, and the operating conditions have not been fully considered. Inspired by the works of similar electrochemical systems, such as Li–ion batteries and hydrogen fuel cells [21,22,23], this paper presents a health status assessment and RUL prediction approach for μDMFC under different operating conditions, where a single degradation metric, such as the output voltage, no longer provides accurate RUL prediction, even when observed continuously. Although the polarization curves can provide accurate health status, such as the internal impedance of the cell, they are difficult to monitor in real time monitored and can only be measured offline at a low frequency. Therefore, this work aims to predict the RUL of the μDMFC by combining external observation with an analysis of the internal characteristics that moderate the environmental impacts on degradation state estimation. Furthermore, different operating conditions are considered in the degradation model to further improve prediction accuracy.

The paper is organized as follows: The preparation and experimental settings of the μDMFC are described in Section 2. Then, the degradation mechanisms and models are explained in Section 3. The implementation of μDMFC RUL prediction and its performance evaluation are presented in Section 4. Section 5 concludes the study.

## 2. μDMFC Preparation and Aging Experiment

**Figure 1:**
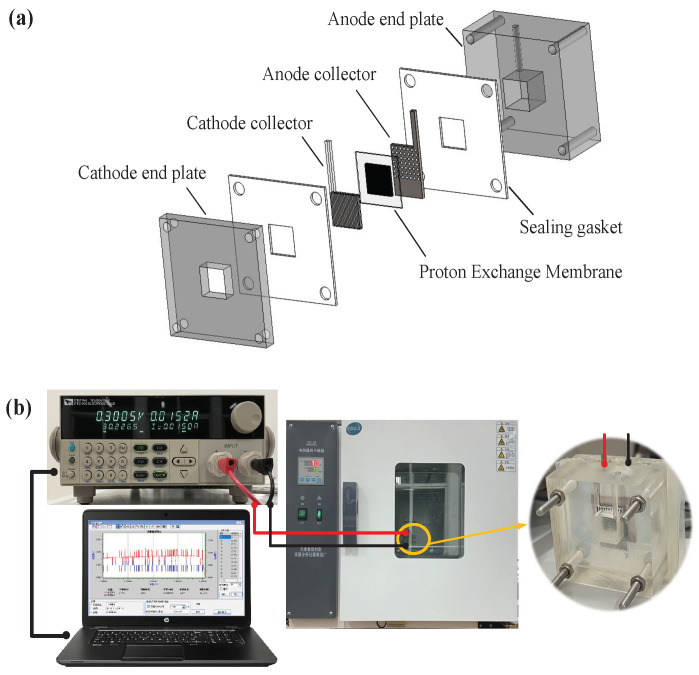
μDMFC aging test: (**a**) Schematic diagram of μDMFC structure; (**b**) μDMFC testing platform.

μDMFC consists of end plates, collector plates, sealing gaskets and membrane electrodes assembly (MEA). The μDMFC structure developed in this work is shown in Figure 1a and its testing platform is illustrated in Figure 1b.

The end plates are made of acrylic, and the anode end plate is 40×40×20 mm${}^{3}$, with a 10×10×10 mm${}^{3}$ reservoir to store the reactants. The cathode end plate is 40×40×5 mm${}^{3}$ with a 10×10 mm${}^{2}$ square hole. The sealing gasket material is silica gel. The collector plate is made of stainless steel, where the electrochemical reaction between methanol and oxygen occurs. The carbon paper is used as the gas diffusion layer for the cathode and the anode of the membrane electrode. The cathode catalyst is of 40 wt% Pt/C (load: 2 mg/cm${}^{2}$), while the anode catalyst is of 60 wt% PtRu/C (load: 2 mg/cm${}^{2}$). The MEA is assembled at 135 °C under 1 MPa, and ultrasonically cleaned with deionized water. The encapsulated μDMFC reservoir cavity was filled with a methanol solution with a concentration of 2 mol/L, and the cell was connected to an electronic load, and placed in a constant temperature and humidity chamber for activation (70 °C for 3 h at a high current). The polarization curve test started from current density of zero and increases discharge at a 10 mA gradient.

**Figure 2:**
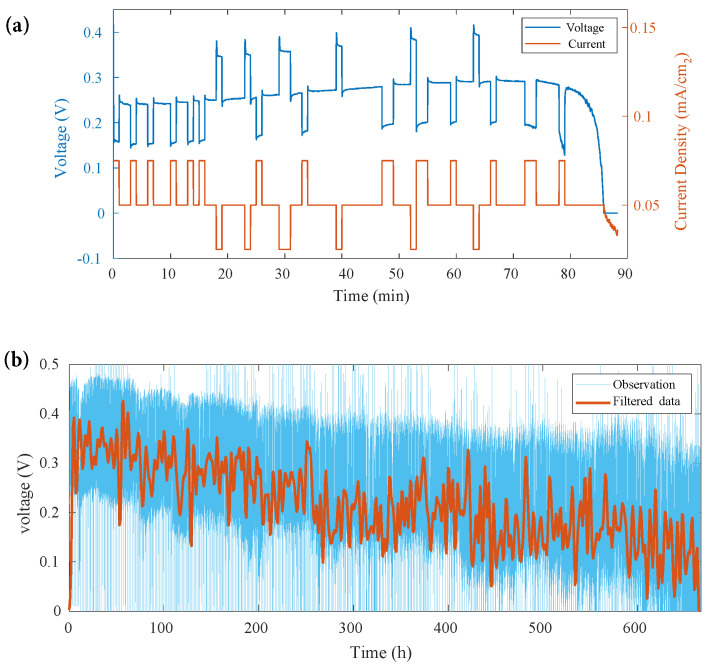
μDMFC output voltage: (**a**) A discharging cycle with loading current; (**b**) collected output voltage and filtered data in aging test.

In this work, the operating temperature is maintained at its normal working temperature of 70 °C to accelerate aging. A discharge cycle is shown in Figure 2a. For μDMFC aging acceleration, three different discharge currents, 25 mA, 50 mA, and 75 mA, were configured to represent ad hoc operating conditions. Fifty milliamps represents normal operation, accounting for about 70% during the whole life service. The randomness of the loading profile is realized by simulating a Markov process. The duty cycles were expressed using the state transition probability of the three states in the Markov chain.

The single-cell μDMFC was tested under the above conditions for about 600 h to its end of life (EOL). The methanol solution was refilled every 90 min once exhausted. Observations of the μDMFC output voltage are shown in Figure 2b. The raw measurement data were filtered using a low-pass filter to eliminate the noise; the degradation trend, caused by aging and fluctuations introduced by loading variations and refilling reactants, were kept. Moreover, when there was a sudden change in current, a positive shock to the voltage occurred. The voltage increased slowly and then decreased sharply. The heat was generated when the electrochemical reaction started inside the cell, resulting in improved discharging and higher voltage. The methanol in the reservoir chamber continued to be consumed and led to a voltage drop. When the methanol was depleted, the cell was refilled.

## 3. μDMFC Degradation Modeling and RUL Prediction Method

### 3.1. μDMFC Degradation Mechanism

μDMFC degradation occurs in multiple components of the cell, including the plates, electrodes, and membranes, and involve various physicochemical processes (chemical, electrochemical, mechanical, and thermodynamic). For a typical μDMFC, CH${}_{3}$OH is fed to the anode; the reaction occurs in the presence of an anode catalyst and produces CO${}_{2}$, where $e^{-}$ and H${}^{+}$ are released. The reaction is as follows:

(1) $${\mathrm{CH}}_{3}\mathrm{OH}+\frac{3}{2}O_{2}\to {\mathrm{CO}}_{2}+2H_{2}O$$

The voltage of the μDMFC was determined using Gibbs Free Energy:

(2) $$E_{r}=-\frac{\Delta G_{r}}{nF}=\varphi_{eq,c}-\varphi_{eq,a}$$

where $E_{r}$ is the maximum voltage of the μDMFC, i.e., the equilibrium potential difference between the cathode and anode of theoretical value of 1.183 V. $\Delta G_{r}$ represents Gibbs Free Energy. *n* is the number of electrons gained or lost in the electrode reaction. *F* is the Faraday Constant. $\varphi_{eq,c}$ is the cathode electric potential of 1.229 V. $\varphi_{eq,a}$ is the anode electric potential of 0.046 V.

**Figure 3:**
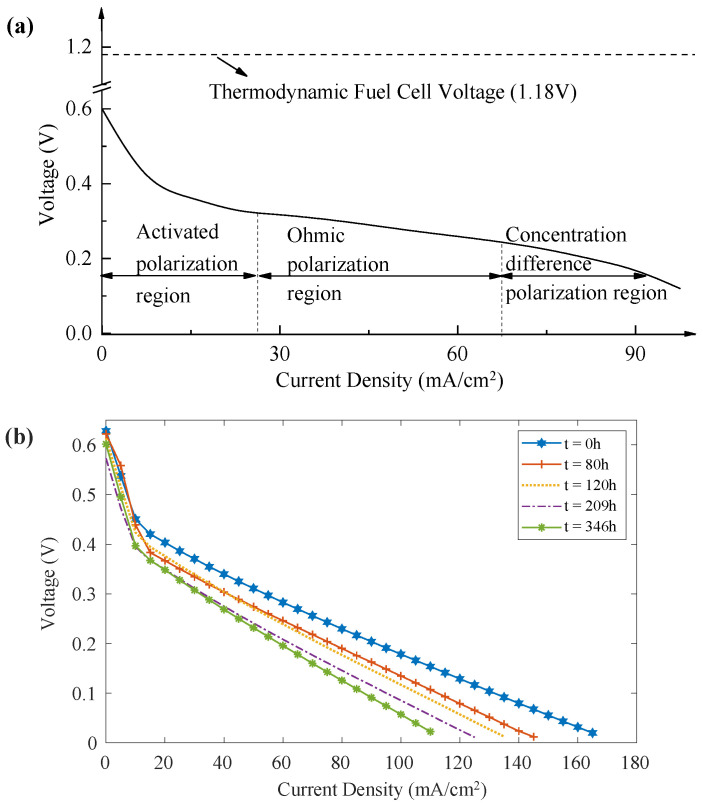
Polarization curves: (**a**) A typical polarization curve of μDMFC; (**b**) Measured polarization curves at different time.

The measured open-circuit voltage (OCV) of the cell in this test is 0.6 V, which is lower than the theoretical value. The difference between the theoretical potential and the actual open-circuit voltage is the open-circuit loss, mainly caused by two factors: First, sub-optimal operating pressure, temperature, and methanol solution concentration in the fuel cell. The activity of the catalyst also restricts the reaction rate, resulting in an OCV lower than the theoretical value. Second, due to the resistance of MEA and the collector plate, which affects the contact resistance between different components, the inside resistance partial voltage of the fuel cell also leads to an OCV lower than the theoretical value. A typical polarization curve is illustrated in Figure 3a [24]; the voltage loss in a single cell is caused by three polarization phenomena: activation polarization, ohmic polarization, and concentration polarization.

In the activated polarization region, the voltage decreases drastically as the current density increases, mainly due to the activation loss of the catalyst, the diffusion transfer resistance within the diffusion layer, and the transfer resistance of the charge through the membrane, etc. [19,25]. The activation polarization overpotential expression is expressed by:

(3) $$\eta_{act}=b\ln\left(\frac{j}{j_{0}}\right)$$

where $\eta_{act}$ is the activation polarization overpotential, $j_{0}$ is the reference current density and *j* represents the current density values for arranging flow channels in fuel cells (mA/cm${}^{2}$).

The dominant resistance in the ohmic polarization region is ohmic loss, in which the curve decreases linearly, which is caused by the conduction resistance of ions and electrons. The ohmic polarization over-potential is expressed by:

(4) $$\eta_{ohm}=jR$$

where $\eta_{ohm}$ is ohmic polarization over potential, *R* is unit area specific resistance.

The concentration polarization region is a high current density interval where the concentration loss dominates. The consumption of methanol in this interval exceeds the mass transfer rate of the cell. The lack of reactants in the electrode and the accumulation of products leads to a sharp decline in the output voltage. The current range of the μDMFC prepared in this work does not locate within this high current interval. The mass transfer loss can thus be neglected. Therefore, the output voltage is expressed by:

(5) $$E_{cell}=E_{r}-\left(\eta_{\mathrm{act}\;}+\eta_{\mathrm{ohm}\;}\right)$$

where $E_{cell}$ is the output voltage. The polarization curve representing the voltage loss can be described by:

(6) $$E_{cell}=E_{r}-b\ln\left(\frac{j}{j_{0}}\right)-jR$$

which can be simplified as:

(7) $$E_{cell}=E_{r}-b\ln\left(aj\right)-jR$$

Therefore, the μDMFC internal parameters $E_{r}$, *R*, *a*, and *b* are identified. When the polarization curves are obtained at different life stages, time-dependent parameter values can be addressed. Figure 3b depicts the polarization curves evolution with aging. It shows that with the progress of the aging test, the maximum discharge current gradually decreases and the fuel cell output voltage decreases.

### 3.2. Particle Filtering-Based RUL Prediction

The output voltage (power) is the most used degradation indicator in fuel cell life assessments because it is convenient to observe. The voltage decreases gradually during the life cycle, and the voltage drops are categorized as reversible or irreversible. Irreversible degradation reflects the cell aging effect, whereas the reversible phenomena may be caused by the operating condition and cell recovery, which are difficult to access directly [26]. The purpose of this work is to estimate the μDMFC health state considering both irreversible and reversible degradation; reversible degradation will be managed by integrating the mechanism explained in Section 3.1.

During RUL prediction, degradation behavior can be learned when the measurements are available at the learning phase using prognostic techniques, for example, Particle Filtering (PF), which has been widely adopted for the degradation path estimation and the prognostics [27,28]. Bayesian estimation techniques have proven capable of treating uncertainties in processes [29]. In this study, we integrate Bayesian estimation into prognostics, accounting for linearity or Gaussian noises.

To this end, we developed the following discrete-time state transition model for describing the degradation dynamics and observations:

(8) $$x_{t}=f_{t}\left(x_{t-1},\omega_{t-1},\Theta_{t-1}\right)$$

(9) $$z_{t}=h_{t}\left(x_{t},\nu_{t}\right)$$

where *t*, *x*, *z*, *f*, and *h* are the system state, the measurement, the degradation model (state transition function) and the measurement model, respectively. ω is the system noise, assumed to be distributed from a Gaussian distribution $\omega_{t}∼N\left(0,{\sigma_{\omega }}_{t}^{2}\right)$. ν is the measurement noise assumed to be sampled from a Gaussian distribution $\nu_{t}∼N\left(0,{\sigma_{\nu }}_{t}^{2}\right)$. Θ is the vector of model parameters.

In this work, the most used state transition model for electrochemical devices degradation trends is selected to propagate the particles for PF:

(10) $$x_{k}=x_{k-1}\cdot e^{-\beta \left(t_{k}-t_{k-1}\right)}$$

where β is the model parameter, $t_{k}$ is the current time step and $t_{k-1}$ is the previous time step.

The probability distribution of the system state is estimated according to the sampled particles and their associated weights. The Bayesian approach is, then, processed to propagate and update the probabilistic information on the unknown states and the model parameters. The algorithm is described as follows.

1. Adopt the model described in Equation (10) to propagate i=1,…,n particles, indicating the probability density function (PDF) of the system states $x_{t-1}$, $x_{t}$;
2. Receive an online measurement $z_{k}$, calculate its likelihood, in the context of the associated weight of each $i^{th}$ particle;
  (11) $$\begin{array}{c} \begin{array}{c} L\left(z_{t}|x_{t}^{i},{\sigma_{\nu }}_{t}^{i}\right)=\frac{1}{\sqrt{2\pi }{\sigma_{\nu }}_{t}^{i}}exp\left[-\frac{1}{2}{\left(\frac{z_{t}-x_{t}^{i}}{{\sigma_{\nu }}_{t}^{i}}\right)}^{2}\right] \end{array} \end{array}$$
3. Given weight limits, delete the particles with small weights and replicate those with large weights by resorting to resampling [30];
4. Buil the posterior PDF, being the prior of the next iteration.

The steps repeat sequentially, and stop when there no measurements are available. The stop time is estimated to be the prediction time $t_{\lambda }$.

**Figure 4:**
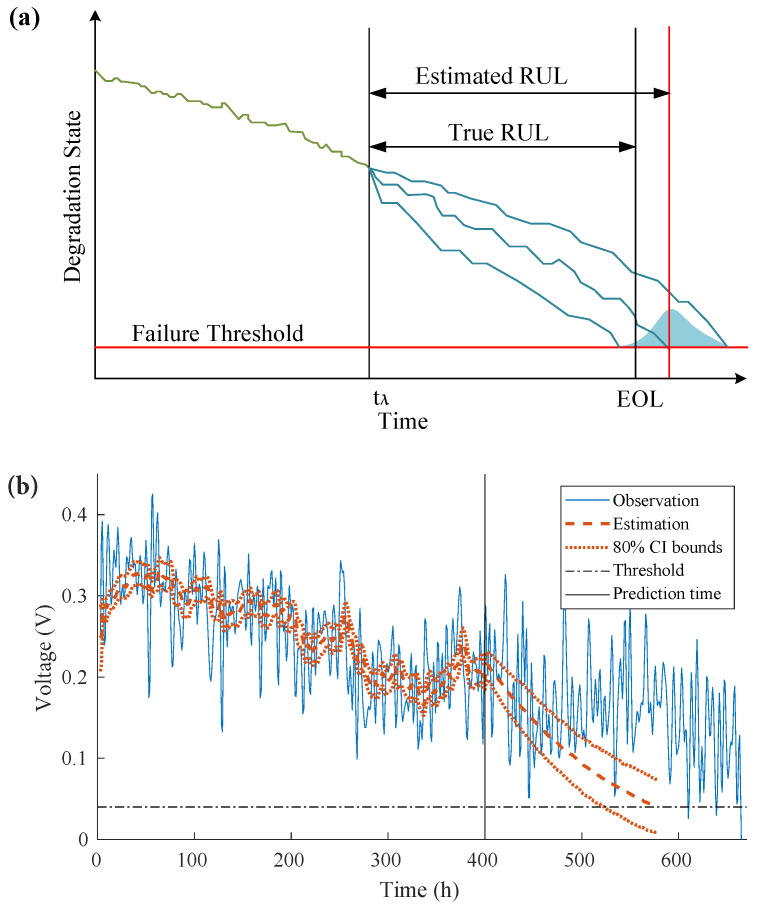
Degradation estimation and RUL prediction: (**a**) illustration of RUL prediction; (**b**) direct observation-based RUL prediction for μDMFC at $t_{\lambda }=400$ h.

The PDF of RUL is obtained when the particles reach the preset failure threshold (FT), as shown in Figure 4a.

In this case, the FT is defined as the 10% of the μDMFC initial output voltage. The procedures of the PF algorithm applied for prognostic purpose [28] are summarized in Algorithm A1, Appendix A.

## 4. Application to μDMFC

External and internal measurements were taken after the μDMFC was prepared. The output voltage (external) is continuously monitored, while the polarization tests (internal) were performed every 24 h. The degradation model parameters identified from the intrusive internal measurements were used to predict the RUL, and the result was compared with that obtained from the degradation trend model.

### 4.1. RUL Prediction Based on Output Voltage

The degradation state transition model is updated by each incoming measurement. When the prediction time is reached, the prediction is propagated through the updated state transition model. An RUL prediction at the prediction time $t_{\lambda }=400$ h is shown in Figure 4b.

The results show that the degradation estimation at the learning stage is consistent with the degradation trend. During the prediction stage, the degradation trend is no longer tolerated because the new measurements are lacking, and the variations in operating conditions are no longer considered. The predicted RUL value was 186 h with an 80% confidence interval (CI) (129–230 h), whereas the actual RUL was 210 h.

### 4.2. RUL Prediction Based on Internal Degradation Model Parameters

#### 4.2.1. Model Parameters Identification

**Figure 5:**
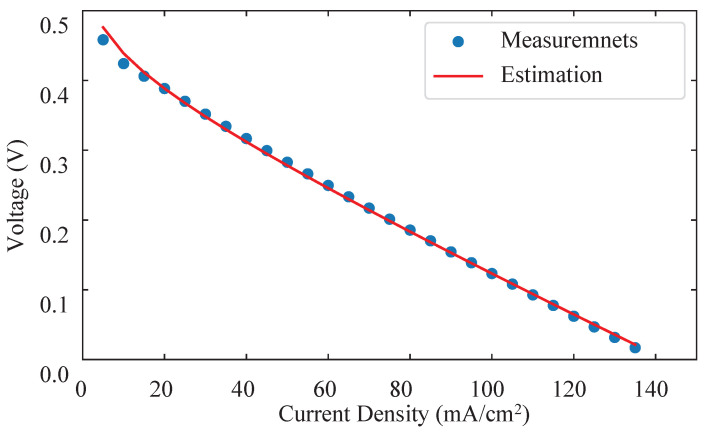
Parameters identification with polarization curve at *t* = 80 h.

The internal parameters of the degradation model can be identified from the polarization curves using the degradation model described in Equation (7). Figure 5 shows a fitting example of model parameter identification with the nonlinear regression technique.

The internal model parameters identified at different time steps are listed in Table 1.

Then, the time evolution of the internal parameters *a*, *b*, and *R* in the degradation model can be predicted by applying the PF algorithm. Initially, the four most utilized functions for electrochemical device degradation trends, i.e., linear, polynomial, logarithmic and experiential, were evaluated for their fit. Based on their respective fitting accuracy, the exponential function was chosen for parameter *a* and *R* evolution, and the polynomial function was chosen for parameter *b* evolution. They are described by:

(12) $$a_{k}=a_{k-1}e^{\beta_{1}\Delta t}$$

(13) $$b_{k}=b_{k-1}\left(1+\beta_{2}\Delta t\right)$$

(14) $$R_{k}=R_{k-1}e^{\beta_{3}\Delta t}$$

**Figure 6:**
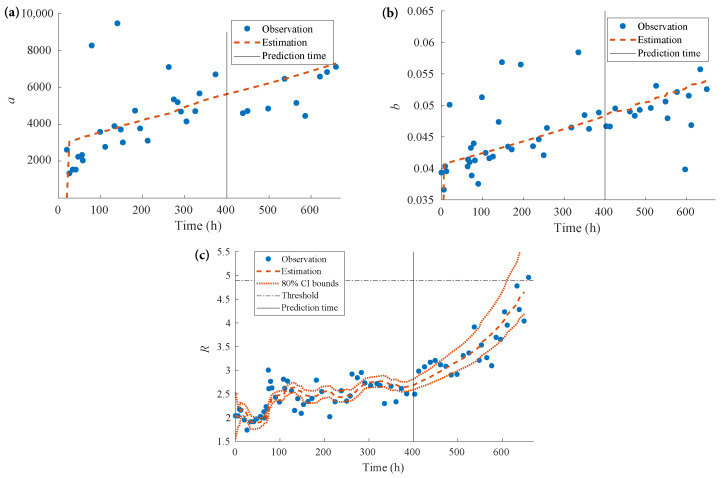
Internal Parameters Prediction at $t_{\lambda }=400$ h: (**a**) Prediction of parameter *a*; (**b**) Prediction of parameter *b*; (**c**) Prediction of parameter *R*.

The examples of parameters prediction at prediction time $t_{\lambda }=400\;h$ are shown in Figure 6a–c.

#### 4.2.2. Operating Condition Estimation

During the accelerated aging test, a current loading profile was simulated by a three-level Markov process. The prediction of the current density *j* was realized by estimating the probability transfer matrix with the usage behavior, i.e., the amplitude of each level in the total 400 h historical data.

The proportion of single discharge state to the total state, i.e., the frequency of each operating condition, can be recorded until $t_{\lambda }=400$ h. During the 400 h learning stage, the duration of each current state was recorded; this allows the proportion of each state to be calculated as follows:

(15) $$p_{i}=\frac{n_{i}}{t_{\lambda }}$$

where $p_{i}$ is the probability of the presence of each loading current level, $n_{i}$ is the total number of steps in the level, and $t_{\lambda }$ is the prediction time step. The probabilities calculated from 400 h of historical data are $p_{1}=0.184,\;p_{2}=0.675,\;p_{3}=0.141$.

The previously calculated $p_{i}$ is, thus, used for the Markov probability transfer metrics to obtain the loading current state for the next steps.

(16) $$P_{k+1}=\left[\begin{array}{ccc} p_{k,1} & p_{k,2} & p_{k,3}\\ p_{k,1} & p_{k,2} & p_{k,3}\\ p_{k,1} & p_{k,2} & p_{k,3} \end{array}\right]$$

**Figure 7:**
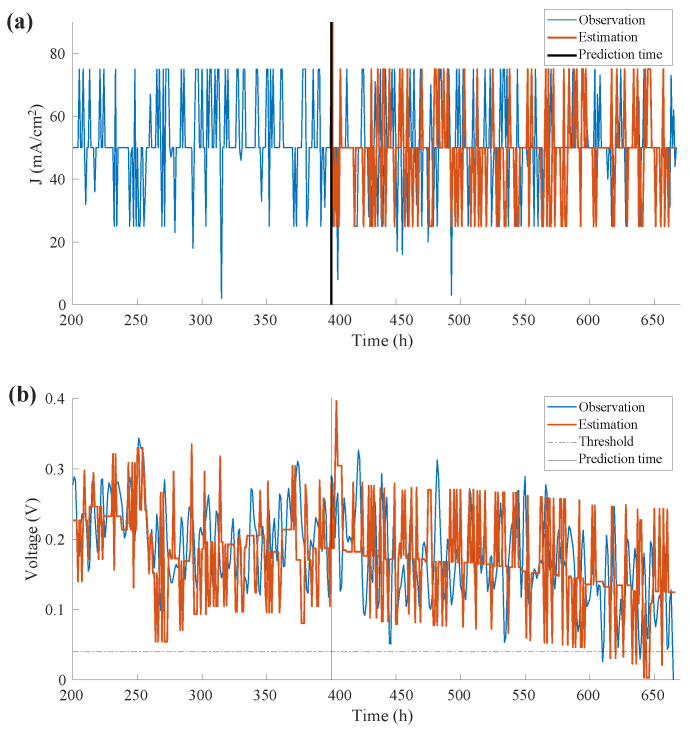
Prediction results: (**a**) Prediction of parameter *I* at $t_{\lambda }=400$ h; (**b**) Degradation Model-based RUL Prediction at $t_{\lambda }=400$ h.

Each probability $P_{k+1}$ is derived from the state transfer matrix of the previous probability $P_{k}$. The first element is derived from the state transfer matrix of the last element in the known data sequence, e.g., the current values of first 400 h. The next probability is not independent of the previous loading current state, but is independent of the probability of the occurrence of each state. Figure 7a shows loading current density predicted at $t_{\lambda }=400$ h based on the usage feature learned until $t_{\lambda }$.

#### 4.2.3. Prediction Results

The predicted values of the parameters *a*, *b*, *R*, and *J* were taken into the degradation model described by Equation (7), and the outcome is the voltage value, as shown in Figure 7b. The output voltage degradation trend and its variations have been well captured by integrating the loading current profile in the learning phase. The degradation behavior captured in the prediction phase is also visible.

To further evaluate the quality of prognostic results, the predictions are made from $t_{\lambda }=300$ h at every 10 h to its EOL of 600 h.

**Figure 8:**
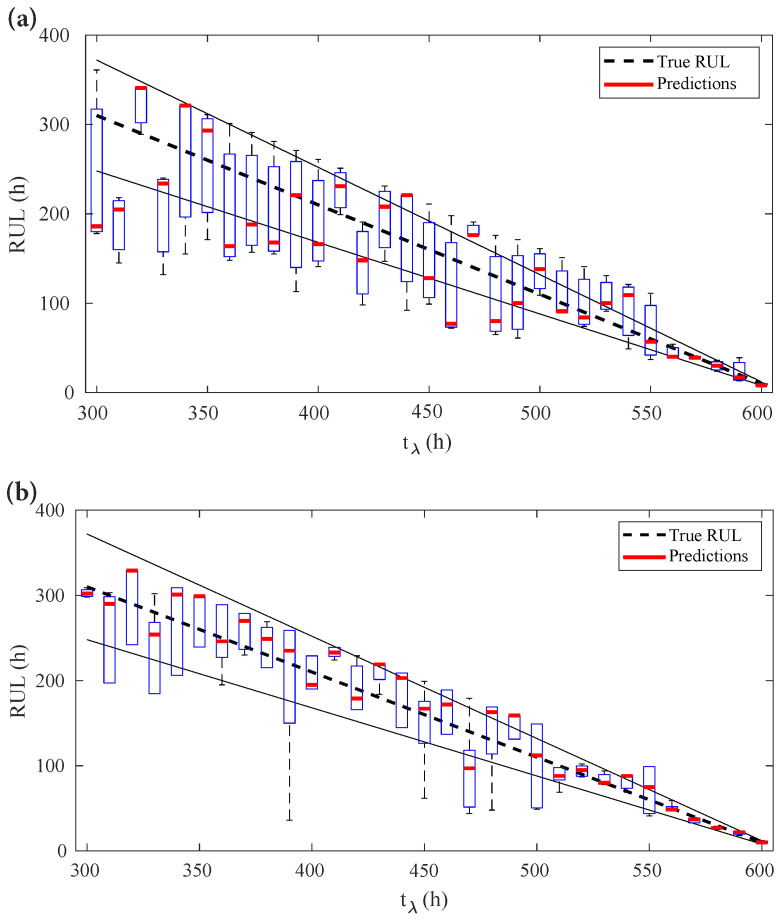
RUL prediction results from 300 to 600 h: (**a**) Output voltage observation-based RUL prediction results; (**b**) Degradation model-based RUL prediction results.

The box-plot diagram in Figure 8a shows the output voltage observation-based RUL prediction results from 300 h to 600 h with an interval of 10 h. The boxplot diagram in Figure 8b shows the internal parameters-based RUL prediction results from 300 h to 600 h with an interval of 10 h. The black dotted line is the true RUL, theoretically calculated by

(17) $$RUL_{\lambda }^{*}=EOL-t_{\lambda }$$

The two solid lines form a shrinking accuracy zone with ±10% of the truth $RUL_{\lambda }^{*}$. Figure 8a,b show that, at the early prediction steps, the predicted RULs are biased from its true values, especially for the predictions made through the direct observation shown in Figure 8a. With the accumulation of learning information, the predictions become more accurate and less uncertain. It can be seen that the degradation-parameters-based model provides better outcomes in these examples. To further validate the models, the RUL prediction results were evaluated by the prognostic performance metrics.

### 4.3. Prediction Quality Evaluation

The prognostic performance indexes of *accuracy*, *precision* and *coverage* are applied to evaluate the prediction quality [28].

The *accuracy* index, Acc, is described by:

(18) $$Acc_{\lambda }=1-\frac{\left|RUL_{\lambda }^{*}-\hat{RUL_{\lambda }}\right|}{RUL_{\lambda }^{*}}$$

where $Acc_{\lambda }$ is the relative accuracy of the prediction step λ, $RUL_{\lambda }^{*}$ and $\hat{RUL_{\lambda }}$ are the true value and predicted value of the RUL, respectively.

The *precision* index, Prc, calculates the relative width between the predicted bounds of the CI:

(19) $$Prc_{\lambda }=\frac{CI^{+}-CI^{-}}{RUL_{\lambda }^{*}}$$

where $CI^{+}$ and $CI^{-}$ are the upper and lower bounds of CI. The smaller the $Prc_{\lambda }$ value, the greater the precision.

The *coverage* index Cvg tests if the real RUL is covered within the RUL prediction CI:

(20) $$Cvg_{\lambda }=CI^{-}\leq RUL_{\lambda }^{*}\leq CI^{+}$$

The closer the value $Cvg_{\lambda }$ is to CI, the better uncertainty is managed.

In this study, three models of two different types are considered:

1. Prediction Model 1, based on the direct observation of output voltage;
2. Prediction models based on the internal degradation parameters with different loading current management:
  - Model 2 with internal parameters considering the loading current of average level (50 mA) during the prediction,
  - Model 3 with internal parameters and the dynamic loading current estimation.

Evaluations of each model’s results are shown in Table 2. The values of those indexes are the average values of 31 predictions evaluated from $t_{\lambda }=300$ to $t_{\lambda }=600$ h with an interval of 10 h.

As the average prediction accuracy of Model 2 is lower than that of Model 1, it can be assumed that, for Model 1, the loading current is not considered specifically for prediction, but the external degradation behavior, to some extent, can be captured by a PF-based approach. Although the loading current was considered in the prediction phase by Model 2, a static value is insufficient for describing operating conditions. Among all compared models, the proposed degradation-parameter-based Model 3 provides the best prognostic performance in terms of all indexes.

## 5. Conclusions

The purpose of this study was to improve RUL prediction quality for the μDMFC under dynamic operating conditions. The following points have been investigated: (1) the impact of operating conditions has been handled using the time evolution of internal degradation parameters estimated from the polarization curves; (2) the integration of an operating conditions prediction mechanism improved RUL prediction quality, and the proposed model outperforms all tested models; and (3) the integration of the internal characterization into RUL prediction demonstrates promising prognostic performance on the experimental dataset considered in this paper. In future work, the proposed approach will be tested and validated by broadening its applications to other available data; information regarding the internal characterization of RUL prediction will also be investigated.

## Figures and Tables

**Table 1:** Degradation model parameters identification results.

Time	*a*	*b*	*R*
0 h	2610	0.050	1.95
80 h	2033	0.06	2.01
120 h	3580	0.051	2.33
209 h	9459	0.047	2.41
346 h	3764	0.056	2.55

**Table 2:** Prognostic performance evaluation results.

Degradation Indicator	Model	Acc	Prc	Cvg
Direct observation	Model 1	0.740	0.578	0.516
Internal parameters	Model 2	0.465	0.305	0.387
	Model 3	**0.803**	**0.245**	**0.806**

## Data Availability

Not applicable.

## Abbreviations

    The following abbreviations are used in this manuscript:

μDMFC	Micro-Direct-Methanol Fuel Cell
MEMS	Micro-Electro Mechanical Systems
RUL	Remaining Useful Life
PEMFC	Proton-Exchange-Membrane Fuel Cell
PHM	Prognostics and Health Management
MEA	Membrane Electrodes Assembly
EOL	End Of Life
OCV	Open-Circuit Voltage
PF	Particle Filtering
CI	Confidence Interval
PDF	Probability Density Function
FT	Failure Threshold

## Appendix A. Particle Filtering-Based RUL Prediction Algorithm

**Algorithm A1** Particle Filtering-based RUL Prediction.
1: Draw particles $x_{0}^{i},{\sigma_{\omega }}_{0}^{i},{\sigma_{\nu }}_{0}^{i}$ and $\Theta_{0}^{i}$ from initial uniform distributions 2: Time step t=1 3: **while** $x_{t}^{i}>FT$ and $t\leq t_{\lambda }$ 4: **for** i=1,…,n // Importance sampling: 5: Draw particles $x_{t}^{i}∼p\left(x_{t}^{i}|x_{t-1}^{i},{\sigma_{\omega }}_{t-1}^{i},\Theta_{t-1}^{i}\right)$ by Equation (10) 6: Assign weight $w_{t}^{i}=L\left(z_{t}|x_{t}^{i},{\sigma_{\nu }}_{t}^{i}\right)$ by Equation (11) 7: **end for** 8: Normalize weight $w_{t}^{i}=w_{t}^{i}/\sum_{i=1}^{n}w_{t}^{i}$ 9: Calculate the cumulative sum of normalized weights: ${\left\{Q_{t}^{i}\right\}}_{i=1}^{n}=Cumsum\left({\left\{w_{t}^{i}\right\}}_{i=1}^{n}\right)$ 10: **for** i=1,…,n // Multinomial Resampling: 11: j=1 12: Draw a random value $u^{i}∼U\left(0,1\right]$ 13: **while** $Q_{t}^{j}<u^{i}$ 14: j=j+1 15: **end while** 16: Update $x_{t}^{i}=x_{t}^{j}$, ${\sigma_{\omega }}_{t}^{i}={\sigma_{\omega }}_{t}^{j},{\sigma_{\nu }}_{t}^{i}={\sigma_{\nu }}_{t}^{j}$, $\Theta_{t}^{i}=\Theta_{t}^{j}$ 17: **end for** 18: t=t+1 19: **end while** // RUL prediction: 20: $t=t_{\lambda }$ // Start from the prediction time 21: **for** i=1,…,n 22: **while** $x_{t}^{i}>FT$ 23: t=t+1 24: Predict particles’ paths $x_{t}^{i}=f\left(x_{t-1}^{i},{\sigma_{\omega }}_{t-1}^{i},\Theta_{t-1}^{i}\right)$ 25: **end while** 26: Estimate ${\hat{RUL}}_{t}^{i}=t-t_{\lambda }$ 27: **end for**
